# 4-[(*E*)-(2,4,5-Trimeth­oxy­benzyl­idene)amino]-1,5-dimethyl-2-phenyl-1*H*-pyrazol-3(2*H*)-one

**DOI:** 10.1107/S1600536810021586

**Published:** 2010-06-16

**Authors:** Hoong-Kun Fun, Madhukar Hemamalini, Abdullah M. Asiri, Salman A. Khan

**Affiliations:** a X-ray Crystallography Unit, School of Physics, Universiti Sains Malaysia, 11800 USM, Penang, Malaysia; b Department of Chemistry, Faculty of Science, King Abdu Aziz University, Jeddah, Saudi Arabia

## Abstract

The title compound, C_21_H_23_N_3_O_4_, adopts an *E* configuration about the central C=N double bond and the pyrazolone ring is almost planar, with a maximum deviation of 0.042 (1) Å. The central pyrazolone ring makes dihedral angles of 51.96 (5) and 3.82 (5)° with the attached phenyl and the trimeth­oxy-substituted benzene rings, respectively. The dihedral angle between the phenyl ring and the trimeth­oxy-substituted benzene ring is 50.19 (5)° and an intra­molecular C—H⋯O hydrogen bond generates an *S*(6) ring motif. The crystal structure is stabilized by inter­molecular C—H⋯O and C—H⋯N hydrogen bonds.

## Related literature

For background to the applications of Schiff bases, see: Vukovic *et al.* (2010 ▶); Ramesh & Maheswaran (2003 ▶); Dongfang *et al.* (2008 ▶); Sastry & Rao (1988 ▶); Kamel *et al.* (2010 ▶). For hydrogen-bond motifs, see: Bernstein *et al.* (1995 ▶). For the stability of the temperature controller used in the data collection, see: Cosier & Glazer (1986 ▶).
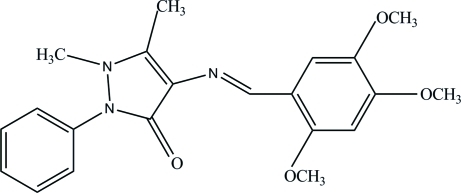


## Experimental

### 

#### Crystal data

C_21_H_23_N_3_O_4_
*M* *_r_* = 381.42
Monoclinic, 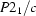

*a* = 21.0128 (10) Å
*b* = 7.4242 (4) Å
*c* = 12.5194 (6) Å
β = 98.675 (1)°
*V* = 1930.72 (17) Å^3^
*Z* = 4
Mo *K*α radiation
μ = 0.09 mm^−1^
*T* = 100 K
0.67 × 0.27 × 0.15 mm

#### Data collection

Bruker APEXII DUO CCD diffractometer
Absorption correction: multi-scan (*SADABS*; Bruker, 2009 ▶) *T*_min_ = 0.941, *T*_max_ = 0.987
23600 measured reflections
5614 independent reflections
4779 reflections with *I* > 2σ(*I*)
*R*_int_ = 0.031

#### Refinement

*R*[*F* ^2^ > 2σ(*F* ^2^)] = 0.040
*wR*(*F* ^2^) = 0.123
*S* = 1.04
5614 reflections
345 parameters
H atoms treated by a mixture of independent and constrained refinement
Δρ_max_ = 0.48 e Å^−3^
Δρ_min_ = −0.23 e Å^−3^

### 

Data collection: *APEX2* (Bruker, 2009 ▶); cell refinement: *SAINT* (Bruker, 2009 ▶); data reduction: *SAINT*; program(s) used to solve structure: *SHELXTL* (Sheldrick, 2008 ▶); program(s) used to refine structure: *SHELXTL*; molecular graphics: *SHELXTL*; software used to prepare material for publication: *SHELXTL* and *PLATON* (Spek, 2009 ▶).

## Supplementary Material

Crystal structure: contains datablocks global, I. DOI: 10.1107/S1600536810021586/hb5480sup1.cif

Structure factors: contains datablocks I. DOI: 10.1107/S1600536810021586/hb5480Isup2.hkl

Additional supplementary materials:  crystallographic information; 3D view; checkCIF report

## Figures and Tables

**Table 1:** Hydrogen-bond geometry (Å, °)

*D*—H⋯*A*	*D*—H	H⋯*A*	*D*⋯*A*	*D*—H⋯*A*
C10—H10*A*⋯O1	0.954 (13)	2.331 (13)	3.0112 (11)	127.8 (10)
C4—H4*A*⋯O1^i^	0.969 (13)	2.541 (13)	3.2628 (12)	131.4 (10)
C20—H20*A*⋯N3^ii^	0.996 (14)	2.577 (14)	3.5383 (13)	162.1 (12)
C20—H20*C*⋯O2^iii^	0.977 (14)	2.509 (14)	3.4470 (13)	160.8 (12)
C20—H20*C*⋯O3^iii^	0.977 (14)	2.495 (15)	3.2779 (13)	137.0 (11)

Symmetry codes: (i) 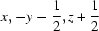; (ii) 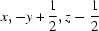; (iii) 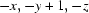.

**Fig. 1.**
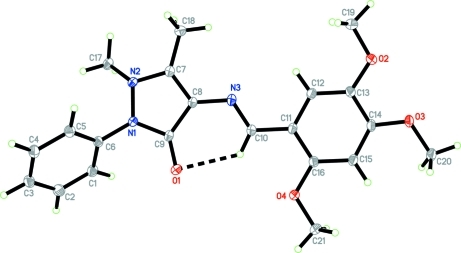
The molecular structure of (I) showing 50% probability displacement ellipsoids. The intramolecular hydrogen bond is shown as a dashed line.

**Fig. 2.**
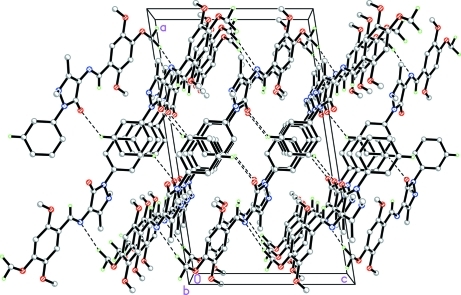
The crystal packing of (I) showing hydrogen-bonded (dashed lines) networks. H atoms not involved in the hydrogen bond interactions are omitted for clarity.

## supplementary crystallographic information

### Comment

Compounds with the structure of AC═NB are known as Schiff base, which can be synthesized from the condensation of amino and active carbonyl groups. Schiff base compounds have shown different therapeutic properties such as antibacterial (Vukovic *et al.*, 2010), antifungal (Ramesh & Maheswaran, 2003), antitumor (Dongfang *et al.*, 2008), anti-inflammatory (Sastry & Rao, 1988) and anticancer activities (Kamel *et al.*, 2010). Due to their importance, the crystal structure determination of the title compound was carried out and the results are presented here.

In the title compound (Fig. 1), the pyrazolone ring (N1/N2/C7–C9) is almost planar, with maximum deviation of 0.042 (1) Å for atom N2. The central pyrazolone (N1/N2/C7–C9) ring makes dihedral angles of 51.96 (5)° and 3.82 (5)° with the attached phenyl ring (C1–C6) and the trimethoxy substituted phenyl ring (C11–C16), respectively. The dihedral angle between the phenyl ring(C1–C6) and the trimethoxy substituted phenyl ring (C11–C16) is 50.19 (5)°. The three methoxy groups are coplanar with the benzene ring [torsion angles C19-O2-C13-C12 = 5.04 (16)°, C20-O3-C14-C15 = -0.36 (14)° and C21-O4-C16-C15 = -1.66 (13)°].

In the crystal packing (Fig. 2), the intramolecular C10—H10A···O1 hydrogen bonding generates an *S*(6) ring motif (Bernstein *et al.*, 1995). The crystal sturcture is futher stabilized by weak intermolecular C4—H4A···O1, C20—H20C···O2, C20—H20C···O3 and C20—H20A···N3 (Table 1) hydrogen bonds.

### Experimental

A mixture of 4-aminophenazone (0.50 g, 0.0033 mol) and 2,4,5-tri-methoxy- benzaldehyde (0.65 g, 0.0033 mol) in methanol (15 ml) was refluxed for 5 h with stirring to give a light yellow precipitate. It was then filtered and washed with methanol to give the pure Schiff base and yellow blocks of (I) were recrystallized from methanol. Yield: 48.18%; Mp. 381°C; IR (KBr) ν_max_ cm^-1^: 2937 (C–H), 1644 (C═C), 1609(C═O), 1591 (C═N), 1122 (N–N). ^1^H-NMR (CDCl_3_) d: 10.02 ((s, 1H, CH olefinic), 7.67 (s, H3, CHaromatic), 6.49 (s, H6, CHaromatic), 7.47–7.26 (m, 5H, CHaromatic), 3.93 (s, OCH_3_), 3.93 (s, OCH_3_), 3.84 (s, OCH_3_), 3.11(s, N-CH_3_), 2.48 (s,-CH_3_).

### Refinement

All the H atoms were located from a difference Fourier map and refined freely [C—H = 0.945 (14)–1.008 (14) Å].

### Crystal data

**Table:**

C_21_H_23_N_3_O_4_	*F*(000) = 808
*M**_r_* = 381.42	*D*_x_ = 1.312 Mg m^−^^3^
Monoclinic, *P*2_1_/*c*	Mo *K*α radiation, λ = 0.71073 Å
Hall symbol: -P 2ybc	Cell parameters from 8559 reflections
*a* = 21.0128 (10) Å	θ = 2.9–34.8°
*b* = 7.4242 (4) Å	µ = 0.09 mm^−^^1^
*c* = 12.5194 (6) Å	*T* = 100 K
β = 98.675 (1)°	Blcok, yellow
*V* = 1930.72 (17) Å^3^	0.67 × 0.27 × 0.15 mm
*Z* = 4	

### Data collection

**Table:**

Bruker APEXII DUO CCD diffractometer	5614 independent reflections
Radiation source: fine-focus sealed tube	4779 reflections with *I* > 2σ(*I*)
graphite	*R*_int_ = 0.031
φ and ω scans	θ_max_ = 30.0°, θ_min_ = 1.0°
Absorption correction: multi-scan (*SADABS*; Bruker, 2009)	*h* = −29→29
*T*_min_ = 0.941, *T*_max_ = 0.987	*k* = −10→10
23600 measured reflections	*l* = −17→17

### Refinement

**Table:**

Refinement on *F*^2^	Primary atom site location: structure-invariant direct methods
Least-squares matrix: full	Secondary atom site location: difference Fourier map
*R*[*F*^2^ > 2σ(*F*^2^)] = 0.040	Hydrogen site location: inferred from neighbouring sites
*wR*(*F*^2^) = 0.123	H atoms treated by a mixture of independent and constrained refinement
*S* = 1.04	*w* = 1/[σ^2^(*F*_o_^2^) + (0.0771*P*)^2^ + 0.3259*P*] where *P* = (*F*_o_^2^ + 2*F*_c_^2^)/3
5614 reflections	(Δ/σ)_max_ < 0.001
345 parameters	Δρ_max_ = 0.48 e Å^−^^3^
0 restraints	Δρ_min_ = −0.22 e Å^−^^3^

### Special details

**Table:**

Experimental. The crystal was placed in the cold stream of an Oxford Cryosystems Cobra open-flow nitrogen cryostat (Cosier & Glazer, 1986) operating at 100.0 (1) K.
Geometry. All s.u.'s (except the s.u. in the dihedral angle between two l.s. planes) are estimated using the full covariance matrix. The cell s.u.'s are taken into account individually in the estimation of s.u.'s in distances, angles and torsion angles; correlations between s.u.'s in cell parameters are only used when they are defined by crystal symmetry. An approximate (isotropic) treatment of cell s.u.'s is used for estimating s.u.'s involving l.s. planes.
Refinement. Refinement of F^2^ against ALL reflections. The weighted R-factor wR and goodness of fit S are based on F^2^, conventional R-factors R are based on F, with F set to zero for negative F^2^. The threshold expression of F^2^ > 2σ(F^2^) is used only for calculating R-factors(gt) etc. and is not relevant to the choice of reflections for refinement. R-factors based on F^2^ are statistically about twice as large as those based on F, and R- factors based on ALL data will be even larger.

### Fractional atomic coordinates and isotropic or equivalent isotropic displacement parameters (Å^2^)

**Table:**

	*x*	*y*	*z*	*U*_iso_*/*U*_eq_	
O1	0.36912 (3)	−0.05337 (10)	0.48796 (5)	0.01654 (15)	
O2	0.02634 (3)	0.14809 (11)	0.14482 (6)	0.02325 (17)	
O3	0.06645 (3)	0.44390 (11)	0.06868 (6)	0.02140 (17)	
O4	0.27842 (3)	0.35928 (10)	0.27787 (6)	0.01722 (15)	
N1	0.35715 (4)	−0.34299 (11)	0.55298 (6)	0.01452 (16)	
N2	0.30506 (4)	−0.45837 (11)	0.56225 (6)	0.01459 (16)	
N3	0.22306 (4)	−0.11616 (11)	0.39236 (6)	0.01426 (16)	
C1	0.47358 (5)	−0.34082 (14)	0.59345 (8)	0.01773 (19)	
C2	0.52970 (5)	−0.35703 (15)	0.66770 (9)	0.0216 (2)	
C3	0.52678 (5)	−0.39395 (14)	0.77564 (8)	0.0205 (2)	
C4	0.46740 (5)	−0.41126 (14)	0.81062 (8)	0.01797 (19)	
C5	0.41087 (5)	−0.39230 (14)	0.73777 (7)	0.01641 (18)	
C6	0.41448 (4)	−0.35847 (13)	0.62948 (7)	0.01427 (18)	
C7	0.25240 (4)	−0.38702 (13)	0.49748 (7)	0.01383 (18)	
C8	0.26770 (4)	−0.22283 (13)	0.45728 (7)	0.01270 (17)	
C9	0.33566 (4)	−0.18791 (13)	0.49592 (7)	0.01293 (17)	
C10	0.24207 (4)	0.03035 (13)	0.35116 (7)	0.01384 (17)	
C11	0.19674 (4)	0.14168 (13)	0.27961 (7)	0.01385 (18)	
C12	0.13242 (4)	0.08699 (14)	0.24831 (7)	0.01533 (18)	
C13	0.08971 (4)	0.18970 (14)	0.17891 (8)	0.01645 (18)	
C14	0.11121 (4)	0.35193 (14)	0.13731 (7)	0.01642 (19)	
C15	0.17427 (4)	0.40938 (14)	0.16780 (7)	0.01589 (18)	
C16	0.21674 (4)	0.30561 (13)	0.24024 (7)	0.01424 (17)	
C17	0.32012 (5)	−0.65126 (14)	0.55935 (8)	0.0194 (2)	
C18	0.18985 (5)	−0.48412 (14)	0.47905 (8)	0.01728 (19)	
C19	0.00170 (5)	−0.00727 (18)	0.19178 (11)	0.0293 (3)	
C20	0.08640 (5)	0.61077 (16)	0.02644 (9)	0.0223 (2)	
C21	0.29988 (5)	0.52608 (14)	0.23842 (8)	0.01750 (19)	
H1A	0.4749 (7)	−0.319 (2)	0.5178 (11)	0.025 (3)*	
H2A	0.5716 (7)	−0.342 (2)	0.6413 (11)	0.027 (4)*	
H3A	0.5674 (7)	−0.413 (2)	0.8265 (12)	0.028 (4)*	
H4A	0.4646 (6)	−0.4380 (19)	0.8855 (11)	0.020 (3)*	
H5A	0.3683 (7)	−0.407 (2)	0.7601 (11)	0.024 (3)*	
H10A	0.2859 (6)	0.0685 (18)	0.3651 (10)	0.016 (3)*	
H12A	0.1206 (7)	−0.025 (2)	0.2767 (11)	0.022 (3)*	
H15A	0.1878 (7)	0.520 (2)	0.1400 (11)	0.020 (3)*	
H17A	0.2798 (7)	−0.723 (2)	0.5642 (11)	0.024 (3)*	
H17B	0.3365 (7)	−0.684 (2)	0.4918 (12)	0.027 (4)*	
H17C	0.3507 (7)	−0.673 (2)	0.6234 (12)	0.030 (4)*	
H18A	0.1555 (7)	−0.411 (2)	0.4352 (12)	0.030 (4)*	
H18B	0.1925 (8)	−0.601 (2)	0.4416 (12)	0.033 (4)*	
H18C	0.1744 (7)	−0.511 (2)	0.5471 (12)	0.032 (4)*	
H19A	−0.0437 (8)	−0.018 (2)	0.1586 (13)	0.037 (4)*	
H19B	0.0243 (8)	−0.119 (2)	0.1720 (13)	0.038 (4)*	
H19C	0.0082 (8)	0.003 (3)	0.2715 (14)	0.042 (4)*	
H20A	0.1208 (7)	0.586 (2)	−0.0182 (11)	0.024 (3)*	
H20B	0.1018 (7)	0.696 (2)	0.0873 (12)	0.032 (4)*	
H20C	0.0475 (7)	0.656 (2)	−0.0181 (11)	0.026 (4)*	
H21A	0.3011 (7)	0.515 (2)	0.1612 (12)	0.023 (3)*	
H21B	0.3418 (7)	0.545 (2)	0.2761 (11)	0.025 (4)*	
H21C	0.2732 (7)	0.626 (2)	0.2555 (11)	0.024 (3)*	

### Atomic displacement parameters (Å^2^)

**Table:**

	*U*^11^	*U*^22^	*U*^33^	*U*^12^	*U*^13^	*U*^23^
O1	0.0170 (3)	0.0133 (3)	0.0192 (3)	−0.0013 (3)	0.0023 (2)	0.0031 (3)
O2	0.0125 (3)	0.0240 (4)	0.0322 (4)	−0.0015 (3)	0.0001 (3)	0.0114 (3)
O3	0.0138 (3)	0.0224 (4)	0.0272 (4)	0.0030 (3)	0.0007 (3)	0.0132 (3)
O4	0.0142 (3)	0.0164 (3)	0.0202 (3)	−0.0017 (3)	−0.0002 (2)	0.0061 (3)
N1	0.0139 (3)	0.0120 (4)	0.0170 (3)	−0.0004 (3)	0.0000 (3)	0.0033 (3)
N2	0.0149 (3)	0.0111 (4)	0.0172 (3)	−0.0007 (3)	0.0004 (3)	0.0030 (3)
N3	0.0153 (3)	0.0137 (4)	0.0137 (3)	0.0033 (3)	0.0020 (3)	0.0020 (3)
C1	0.0173 (4)	0.0173 (5)	0.0189 (4)	0.0029 (4)	0.0038 (3)	0.0031 (4)
C2	0.0151 (4)	0.0213 (5)	0.0281 (5)	0.0019 (4)	0.0027 (4)	0.0041 (4)
C3	0.0190 (4)	0.0159 (4)	0.0243 (5)	0.0007 (4)	−0.0041 (3)	0.0008 (4)
C4	0.0218 (4)	0.0150 (4)	0.0158 (4)	0.0018 (4)	−0.0013 (3)	−0.0005 (3)
C5	0.0173 (4)	0.0151 (4)	0.0166 (4)	0.0019 (3)	0.0020 (3)	−0.0005 (3)
C6	0.0148 (4)	0.0110 (4)	0.0163 (4)	0.0020 (3)	−0.0001 (3)	0.0004 (3)
C7	0.0153 (4)	0.0132 (4)	0.0130 (4)	0.0014 (3)	0.0023 (3)	0.0005 (3)
C8	0.0140 (4)	0.0119 (4)	0.0122 (3)	0.0020 (3)	0.0020 (3)	0.0007 (3)
C9	0.0149 (4)	0.0119 (4)	0.0122 (3)	0.0023 (3)	0.0026 (3)	0.0010 (3)
C10	0.0145 (4)	0.0131 (4)	0.0138 (4)	0.0025 (3)	0.0018 (3)	0.0012 (3)
C11	0.0146 (4)	0.0132 (4)	0.0140 (4)	0.0023 (3)	0.0027 (3)	0.0024 (3)
C12	0.0149 (4)	0.0143 (4)	0.0171 (4)	0.0020 (3)	0.0037 (3)	0.0038 (3)
C13	0.0122 (4)	0.0176 (4)	0.0197 (4)	0.0017 (3)	0.0031 (3)	0.0039 (4)
C14	0.0144 (4)	0.0175 (4)	0.0176 (4)	0.0043 (3)	0.0030 (3)	0.0056 (3)
C15	0.0155 (4)	0.0154 (4)	0.0171 (4)	0.0023 (3)	0.0035 (3)	0.0051 (3)
C16	0.0131 (4)	0.0150 (4)	0.0147 (4)	0.0013 (3)	0.0024 (3)	0.0019 (3)
C17	0.0216 (4)	0.0111 (4)	0.0244 (5)	0.0012 (4)	−0.0003 (4)	0.0034 (4)
C18	0.0166 (4)	0.0155 (4)	0.0196 (4)	−0.0020 (3)	0.0025 (3)	0.0013 (3)
C19	0.0165 (4)	0.0268 (6)	0.0443 (7)	−0.0026 (4)	0.0037 (4)	0.0137 (5)
C20	0.0179 (4)	0.0221 (5)	0.0268 (5)	0.0031 (4)	0.0028 (4)	0.0124 (4)
C21	0.0194 (4)	0.0139 (4)	0.0194 (4)	−0.0017 (4)	0.0036 (3)	0.0029 (3)

### Geometric parameters (Å, °)

**Table:**

O1—C9	1.2341 (12)	C8—C9	1.4603 (12)
O2—C13	1.3709 (11)	C10—C11	1.4613 (12)
O2—C19	1.4268 (14)	C10—H10A	0.954 (13)
O3—C14	1.3583 (11)	C11—C16	1.4011 (13)
O3—C20	1.4342 (13)	C11—C12	1.4090 (13)
O4—C16	1.3698 (11)	C12—C13	1.3808 (13)
O4—C21	1.4311 (12)	C12—H12A	0.949 (15)
N1—C9	1.3937 (12)	C13—C14	1.4130 (14)
N1—N2	1.4084 (11)	C14—C15	1.3897 (13)
N1—C6	1.4261 (11)	C15—C16	1.4029 (12)
N2—C7	1.3754 (11)	C15—H15A	0.953 (15)
N2—C17	1.4683 (13)	C17—H17A	1.008 (14)
N3—C10	1.2927 (12)	C17—H17B	0.990 (14)
N3—C8	1.3918 (11)	C17—H17C	0.963 (15)
C1—C6	1.3898 (13)	C18—H18A	1.000 (16)
C1—C2	1.3923 (13)	C18—H18B	0.990 (17)
C1—H1A	0.965 (14)	C18—H18C	0.977 (15)
C2—C3	1.3893 (15)	C19—H19A	0.986 (17)
C2—H2A	0.992 (15)	C19—H19B	1.006 (17)
C3—C4	1.3892 (15)	C19—H19C	0.990 (18)
C3—H3A	0.995 (15)	C20—H20A	0.996 (14)
C4—C5	1.3913 (13)	C20—H20B	1.007 (16)
C4—H4A	0.969 (13)	C20—H20C	0.976 (15)
C5—C6	1.3921 (13)	C21—H21A	0.974 (14)
C5—H5A	0.984 (14)	C21—H21B	0.945 (14)
C7—C8	1.3753 (13)	C21—H21C	0.972 (15)
C7—C18	1.4863 (13)		
			
C13—O2—C19	116.72 (8)	C13—C12—H12A	122.3 (8)
C14—O3—C20	117.04 (8)	C11—C12—H12A	116.3 (8)
C16—O4—C21	117.65 (7)	O2—C13—C12	125.46 (9)
C9—N1—N2	110.43 (7)	O2—C13—C14	115.26 (8)
C9—N1—C6	125.90 (8)	C12—C13—C14	119.28 (8)
N2—N1—C6	118.94 (7)	O3—C14—C15	124.05 (9)
C7—N2—N1	106.45 (7)	O3—C14—C13	115.62 (8)
C7—N2—C17	121.21 (8)	C15—C14—C13	120.33 (8)
N1—N2—C17	114.72 (8)	C14—C15—C16	119.75 (9)
C10—N3—C8	119.36 (8)	C14—C15—H15A	119.3 (8)
C6—C1—C2	118.90 (9)	C16—C15—H15A	120.9 (8)
C6—C1—H1A	119.6 (8)	O4—C16—C11	116.75 (8)
C2—C1—H1A	121.5 (8)	O4—C16—C15	122.63 (9)
C3—C2—C1	120.62 (9)	C11—C16—C15	120.61 (8)
C3—C2—H2A	121.2 (8)	N2—C17—H17A	109.0 (8)
C1—C2—H2A	118.2 (8)	N2—C17—H17B	111.4 (9)
C4—C3—C2	119.89 (9)	H17A—C17—H17B	108.9 (12)
C4—C3—H3A	120.6 (9)	N2—C17—H17C	105.3 (9)
C2—C3—H3A	119.4 (9)	H17A—C17—H17C	108.8 (12)
C3—C4—C5	120.18 (9)	H17B—C17—H17C	113.3 (12)
C3—C4—H4A	120.8 (8)	C7—C18—H18A	111.7 (9)
C5—C4—H4A	119.0 (8)	C7—C18—H18B	112.7 (9)
C4—C5—C6	119.34 (9)	H18A—C18—H18B	107.7 (13)
C4—C5—H5A	121.7 (8)	C7—C18—H18C	111.5 (9)
C6—C5—H5A	118.9 (8)	H18A—C18—H18C	106.4 (12)
C1—C6—C5	121.05 (8)	H18B—C18—H18C	106.5 (13)
C1—C6—N1	118.69 (8)	O2—C19—H19A	106.4 (10)
C5—C6—N1	120.26 (8)	O2—C19—H19B	110.6 (9)
C8—C7—N2	110.21 (8)	H19A—C19—H19B	106.9 (14)
C8—C7—C18	128.54 (8)	O2—C19—H19C	110.6 (11)
N2—C7—C18	121.25 (8)	H19A—C19—H19C	114.1 (14)
C7—C8—N3	122.95 (8)	H19B—C19—H19C	108.1 (14)
C7—C8—C9	107.87 (8)	O3—C20—H20A	109.0 (9)
N3—C8—C9	129.17 (8)	O3—C20—H20B	110.2 (9)
O1—C9—N1	124.44 (8)	H20A—C20—H20B	111.3 (12)
O1—C9—C8	131.11 (8)	O3—C20—H20C	104.0 (9)
N1—C9—C8	104.37 (8)	H20A—C20—H20C	111.0 (11)
N3—C10—C11	120.57 (8)	H20B—C20—H20C	111.1 (12)
N3—C10—H10A	121.7 (8)	O4—C21—H21A	109.1 (9)
C11—C10—H10A	117.8 (8)	O4—C21—H21B	105.8 (9)
C16—C11—C12	118.58 (8)	H21A—C21—H21B	110.2 (12)
C16—C11—C10	120.31 (8)	O4—C21—H21C	111.1 (8)
C12—C11—C10	121.11 (8)	H21A—C21—H21C	112.6 (12)
C13—C12—C11	121.40 (9)	H21B—C21—H21C	107.8 (12)
			
C9—N1—N2—C7	8.54 (10)	C7—C8—C9—O1	−173.56 (9)
C6—N1—N2—C7	165.59 (8)	N3—C8—C9—O1	5.87 (16)
C9—N1—N2—C17	145.49 (8)	C7—C8—C9—N1	3.25 (10)
C6—N1—N2—C17	−57.45 (11)	N3—C8—C9—N1	−177.33 (9)
C6—C1—C2—C3	1.32 (16)	C8—N3—C10—C11	177.82 (8)
C1—C2—C3—C4	−1.34 (17)	N3—C10—C11—C16	176.26 (8)
C2—C3—C4—C5	0.21 (16)	N3—C10—C11—C12	−4.03 (14)
C3—C4—C5—C6	0.91 (16)	C16—C11—C12—C13	1.10 (14)
C2—C1—C6—C5	−0.17 (15)	C10—C11—C12—C13	−178.61 (9)
C2—C1—C6—N1	−179.57 (9)	C19—O2—C13—C12	5.04 (16)
C4—C5—C6—C1	−0.93 (15)	C19—O2—C13—C14	−175.23 (10)
C4—C5—C6—N1	178.45 (9)	C11—C12—C13—O2	−179.44 (9)
C9—N1—C6—C1	−65.96 (13)	C11—C12—C13—C14	0.83 (15)
N2—N1—C6—C1	140.85 (9)	C20—O3—C14—C15	−0.36 (14)
C9—N1—C6—C5	114.64 (11)	C20—O3—C14—C13	178.96 (9)
N2—N1—C6—C5	−38.55 (13)	O2—C13—C14—O3	−0.49 (13)
N1—N2—C7—C8	−6.30 (10)	C12—C13—C14—O3	179.26 (9)
C17—N2—C7—C8	−139.83 (9)	O2—C13—C14—C15	178.86 (9)
N1—N2—C7—C18	173.92 (8)	C12—C13—C14—C15	−1.40 (15)
C17—N2—C7—C18	40.39 (13)	O3—C14—C15—C16	179.28 (9)
N2—C7—C8—N3	−177.54 (8)	C13—C14—C15—C16	0.00 (15)
C18—C7—C8—N3	2.22 (15)	C21—O4—C16—C11	179.81 (8)
N2—C7—C8—C9	1.93 (10)	C21—O4—C16—C15	−1.66 (13)
C18—C7—C8—C9	−178.31 (9)	C12—C11—C16—O4	176.04 (8)
C10—N3—C8—C7	−174.99 (8)	C10—C11—C16—O4	−4.24 (13)
C10—N3—C8—C9	5.66 (14)	C12—C11—C16—C15	−2.52 (14)
N2—N1—C9—O1	169.88 (8)	C10—C11—C16—C15	177.20 (8)
C6—N1—C9—O1	14.78 (14)	C14—C15—C16—O4	−176.49 (9)
N2—N1—C9—C8	−7.20 (9)	C14—C15—C16—C11	1.98 (14)
C6—N1—C9—C8	−162.30 (8)		

### Hydrogen-bond geometry (Å, °)

**Table:**

*D*—H···*A*	*D*—H	H···*A*	*D*···*A*	*D*—H···*A*
C10—H10A···O1	0.954 (13)	2.331 (13)	3.0112 (11)	127.8 (10)
C4—H4A···O1^i^	0.969 (13)	2.541 (13)	3.2628 (12)	131.4 (10)
C20—H20A···N3^ii^	0.996 (14)	2.577 (14)	3.5383 (13)	162.1 (12)
C20—H20C···O2^iii^	0.977 (14)	2.509 (14)	3.4470 (13)	160.8 (12)
C20—H20C···O3^iii^	0.977 (14)	2.495 (15)	3.2779 (13)	137.0 (11)

Symmetry codes: (i) *x*, −*y*−1/2, *z*+1/2; (ii) *x*, −*y*+1/2, *z*−1/2; (iii) −*x*, −*y*+1, −*z*.
